# Silver Nanoparticle-Embedded Thin Silica-Coated Graphene Oxide as an SERS Substrate

**DOI:** 10.3390/nano6100176

**Published:** 2016-09-22

**Authors:** Xuan-Hung Pham, Eunil Hahm, Hyung-Mo Kim, Seongbo Shim, Tae Han Kim, Dae Hong Jeong, Yoon-Sik Lee, Bong-Hyun Jun

**Affiliations:** 1 Department of Bioscience and Biotechnology, Konkuk University, Seoul 143-701, Korea; phamricky@gmail.com (X.-H.P.); dkhei0525@hanmail.net (E.H.); hmkim0109@konkuk.ac.kr (H.-M.K.); sb201473049@konkuk.ac.kr (S.S.); kth890304@naver.com (T.H.K.); 2 Department of Chemistry Education, Seoul National University, Seoul 151-742, Korea; jeongdh@snu.ac.kr; 3 School of Chemical and Biological Engineering, Seoul National University, Seoul 151-742, Korea; yslee@snu.ac.kr

**Keywords:** graphene oxide, silica coating, surface-enhanced Raman scattering (SERS), signal enhancement

## Abstract

A hybrid of Ag nanoparticle (NP)-embedded thin silica-coated graphene oxide (GO@SiO_2_@Ag NPs) was prepared as a surface-enhanced Raman scattering (SERS) substrate. A 6 nm layer of silica was successfully coated on the surface of GO by the physical adsorption of sodium silicate, followed by the hydrolysis of 3-mercaptopropyl trimethoxysilane. Ag NPs were introduced onto the thin silica-coated graphene oxide by the reduction of Ag^+^ to prepare GO@SiO_2_@Ag NPs. The GO@SiO_2_@Ag NPs exhibited a 1.8-fold enhanced Raman signal compared to GO without a silica coating. The GO@SiO_2_@Ag NPs showed a detection limit of 4-mercaptobenzoic acid (4-MBA) at 0.74 μM.

## 1. Introduction

Surface-enhanced Raman scattering (SERS) has attracted considerable interest for bioanalytical and imaging applications and in environmental monitoring as a non-destructive, ultrasensitive, and selective analytical technique [1,2,3,4,5,6,7,8,9]. SERS enhancement is mediated by chemical and electromagnetic mechanisms of metal substrates, such as Au or Ag [2]. Thus, the metal nanostructure and the SERS substrate material plays an important role in generating strong SERS signals [2,10]. To improve SERS activity, nanomaterials of various sizes, structures, and components, such as nanospheres, nanoshells, nanorods, and multibranched NPs, have been developed and used as SERS probes [4,11,12,13,14,15].

Graphene, a kind of carbon allotrope with a perfect 2D atomic crystal made of sp^2^ carbon atoms, was discovered in 2004 [16]. Graphene has been used as a Raman probe, a substrate, and an additive and building block for SERS on a flat surface [17]. In particular, graphene can be a promising SERS substrate due to its ability to generate strong chemical enhancement [17,18,19,20,21]. Compared with graphene, GO can be produced at a high yield and low cost, which facilitates its practical application [22,23,24,25,26,27,28,29,30,31,32,33,34,35,36,37,38]. However, the chemical enhancement of GO itself exhibits low sensitivity. To overcome this, composites that integrate the advantages of GO and high electromagnetic enhancement of gold or silver nanoparticles (Au or Ag NPs) have been developed for SERS detection [39,40,41,42,43,44,45,46]. Metal NPs were deposited on the surface of GO via oxygen-containing functional groups, such as carbonyl and hydroxyl groups [47,48]. However, a hybrid of GO with Ag NPs can give large background signals from the D and G bands of GO, which can limit their applications [49,50]. Therefore, GO was coated with a silica layer by the hydrolysis of tetraethylorthosilicate (TEOS) or (3-aminopropyl)-trimethoxysilane and applied as a thermally conducting material [51,52,53,54,55]; however, thin-silica-coated GO has not been reported.

In this study, we prepared Ag NP-embedded thin silica-coated GO (GO@SiO_2_@Ag NPs). A thin layer of silica was coated onto the GO surface to enhance SERS signals. Thiol-functionalized thin-silica-coated GO was successfully prepared by physical adsorption of sodium silicate, followed by hydrolysis of 3-mercaptopropyl trimethoxysilane (3-MPTS). Afterwards, Ag NPs were introduced onto the thin silica-coated GO by the reduction of silver nitrate. Herein, a thin layer of silica on the surface of GO played several roles in being the platform to homogeneously immobilize Ag NPs on the surface of GO, reducing the background Raman signal from D and G bands of GO and allowing the Raman reporter close to the surface of GO@SiO_2_@Ag NPs to enhance the Raman signals both from Ag NPs to Ag NPs and from Ag NPs to the GO platform. Subsequently, the resulting GO@SiO_2_@Ag NP was applied as a SERS substrate for the detection of 4-mercaptobenzoic acid (4-MBA). Compared with non-silica-coated Ag-embedded GO, it gave stronger SERS signals.

## 2. Results and Discussion

### 2.1. Preparation of Ag NP-Embedded Silica-Coated GO

**Figure 1:**
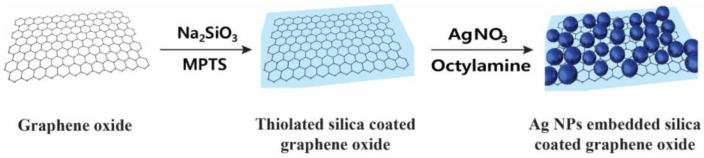
Synthesis of silver nanoparticle-embedded silica-coated graphene oxide.

The fabrication process of the proposed Ag NP-embedded silica-coated GO (GO@SiO_2_@Ag NPs) is illustrated in Figure 1. Ag NPs were embedded on the surface of the thiolated thin silica-coated graphene (GO@SiO_2_), a carrier template, to enhance the SERS. The GO@SiO_2_ was synthesized by introducing a thin silica layer on the surface of GO through the physical adsorption of sodium silicate to the oxygen-contained functional groups as shown in Figure 1 [56]. The generation of a thin silica shell is important for enhancing the SERS [57,58]. 3-MPTS was then used to further generate a thin silica layer with thiol groups, which can strongly interact with Ag NPs in the next step. 

**Figure 2:**
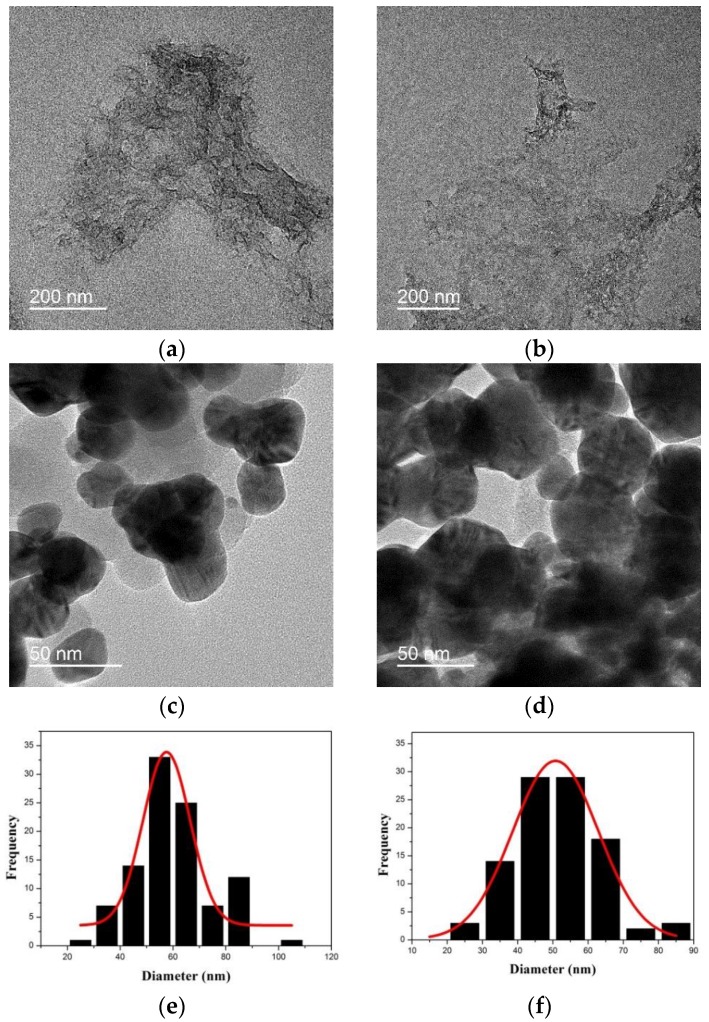
High resolution TEM images of (**a**) graphene oxide; (**b**) thiolated thin silica-coated graphene oxide; (**c**) silver nanoparticle-embedded silica-coated graphene oxide GO@SiO_2_@Ag NPs; and (**d**) silver nanoparticle-embedded graphene oxide (GO@Ag NPs); histograms of (**e**) silver nanoparticle-embedded silica-coated graphene oxide and (**f**) silver nanoparticle-embedded graphene oxide.

Figure 2 shows TEM images of GO, thiolated thin silica-coated GO (GO@SiO_2_), Ag NP-embedded silica-coated GO (GO@SiO_2_@Ag NPs), and Ag NP-embedded GO (GO@Ag NPs). The GO sheet was transparent and wrinkled with several layers (Figure 2a) since GO sheets tend to form a multilayer agglomerate [59]. After modification with sodium silicate and 3-MPTS, GO became thicker and flatter (Figure 2b). This result was confirmed by atomic force microscopy (AFM) using noncontact mode to measure the thickness of GO and GO@SiO_2_ (Figure S1). The thickness of GO was ~8 nm, indicating that GO had several layers instead of a single layer [60]. After coating with silica, the thickness of GO increased to ~20 nm. We assumed that both sides of GO were coated with a silica layer of 6 nm thickness. To confirm the presence of silica layer on the surface of GO, energy-dispersive X-ray spectroscopy (EDS) of sodium silicate-coated GO and thiolated GO@SiO_2_ were recorded in Figure S2. Indeed, sodium silicate-coated GO contained C, O, Si, and Na elements with the atomic composition of 5.5%, 62.0%, 25.3%, and 7.2%, respectively (Figure S2a). After MPTS was deposited on the GO, C and Si element increased to 8.0% and 30.9% while O element slightly decreased to 58.3% (Figure S2b). The presence of 0.3% S element confirmed the successful deposition of 3-MPTS with thiol group on GO@SiO_2_. Ag NPs were embedded on GO@SiO_2_ through the reduction of silver nitrate by octylamine with polyvinylpyrrolidone (PVP) in ethylene glycol [56,61]. The presence of PVP assists the growth and homogeneous distribution of the plasmonic Ag NPs on the surface of GO [62]. Ag NPs were immobilized on the surface of GO@SiO_2_ by the affinity of thiol groups for Ag. Figure 2c shows transmission electron microscope (TEM) images of GO@SiO_2_@Ag. The surface of GO@SiO_2_@Ag was fully covered with Ag NPs. The average size of Ag NPs measured from 100 nanoparticles was 58 ± 18 nm (Figure 2e). Additionally, a part of Ag NPs on the surface of GO@SiO_2_@Ag NPs was merged, forming aggregated structures. Without the thin silica layer, GO was partly covered with Ag NPs, as shown in Figure 2d. The average size of Ag NPs on the surface of GO was estimated to be 51 ± 20 nm, slightly smaller than that of GO@SiO_2_@Ag NPs (Figure 2f). Compared to GO@SiO_2_@Ag NPs, the density of Ag NPs on GO@Ag NPs was slightly lower, likely due to differences in surface properties and the functional groups. In the absence of the silica layer, Ag^+^ was absorbed on the carboxyl groups of GO via electrostatic interaction [63] and was reduced by octylamine. Meanwhile, Ag NPs were synthesized in situ and absorbed on the surface of silica-coated GO via the affinity of Ag NPs and thiol groups (−SH).

The optical properties of GO@SiO_2_@Ag NPs were investigated by UV-VIS spectroscopy. In Figure S2, the UV-VIS spectrum of GO showed a characteristic peak at ~230 nm that corresponded to a π-π* plasmon peak of the sp^2^ carbon of GO [64]. Following silica coating, the intensity of this peak decreased slightly due to the difference in the extinction coefficient. However, no new and significant peak was obtained. The presence of Ag NPs on the surface of GO@SiO_2_ was confirmed by TEM (Figure 2d), and the appearance of a broad absorbance band from 320 to 1000 nm with a maximum peak at ~440 nm. This absorption may be due to the diversity in the size of the Ag NPs, which produces a continuous spectrum of high-order plasmon modes [65,66,67]. In particular, the spectrum intensity of GO@SiO_2_@Ag NPs was increased compared to that of GO@SiO_2_. The spectrum pattern of GO@Ag NPs was similar to that of GO@SiO_2_@Ag NPs, with a broad adsorption peak from 320 to 1000 nm, and a maximum peak of ~454 nm. However, the spectrum intensity of GO@Ag NPs was markedly lower than that of GO@SiO_2_@Ag NPs (Figure S3).

### 2.2. SERS Activity of GO@SiO_2_@Ag NPs

**Figure 3:**
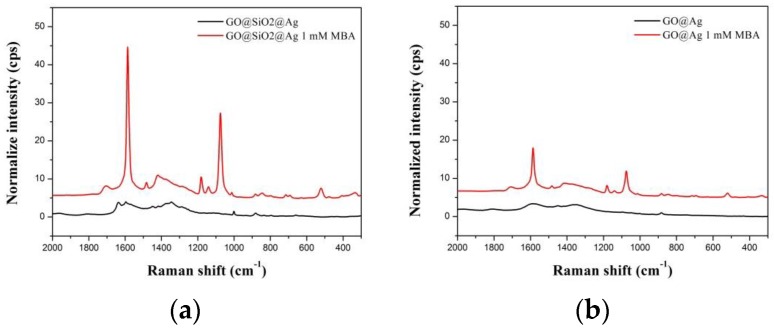
SERS spectra of (**a**) GO@SiO_2_@Ag NPs and (**b**) GO@Ag NPs in EtOH solution with and without 1 mM 4-mercaptobenzoic acid. GO concentration is 1 mg/mL, laser power is 10 mW, wavelength is 532 nm, integration time is 5 s, and laser spot is 2 µm.

The SERS activities of GO@SiO_2_@Ag NPs were investigated using 4-mercaptobenzoic acid (4-MBA) as a SERS chemical. To demonstrate the advantage of GO@SiO_2_@Ag NPs, GO without silica coating was used as a control. Ag NPs were introduced onto the surface of GO by the reported method of Kang et al. [61]. Both the GO@Ag NPs and GO@SiO_2_@Ag NPs were incubated with 4-MBA to immobilize the Raman chemical on their surface. The Raman signals were recorded using a micro Raman system in a capillary tube. Figure 3b shows the SERS spectra of GO@Ag NPs and GO@SiO_2_@Ag NPs in ethanol solution with and without 4-mercaptopropionic acid (4-MBA, 1 mM). In the absence of 4-MBA, the typical broad peaks at ~1342 cm^−1^ and 1595 cm^−1^ were evident, corresponding to the D and G peaks of GO, respectively [17,68]. In the presence of 4-MBA, dominant and distinct 4-MBA peaks were observed from GO@Ag NPs and GO@SiO_2_@Ag NPs at 1072, 1142, 1175, 1370, 1432, 1582, and 1710 cm^−1^ [69,70]. The peaks at 1142 and 1432 cm^−1^ were assigned to the C−H in-plane and out-of-plane bending vibrations. The peaks at 1582 and 1072 cm^−1^ were attributed to ring C−C and C−S stretching, respectively [71]. The weak peak at ~1710 cm^−1^ corresponded to the stretching of the C=O group, while the peak at 1370 cm^−1^ was due to COO-stretching. Therefore, 4-MBA remained as free and the dissociated carboxyl groups on the surface of GO@SiO_2_@Ag NPs. Interestingly, the SERS signal of the GO@SiO_2_@Ag NPs at 1072 cm^−1^ was ~3277 ± 23 cps with 1 mM 4-MBA, and was 1.8-fold greater than that of GO@Ag NPs (1839 ± 419 cps). Similarly, this value was 1.68-fold higher at 1582 cm^−1^. The differences in SERS signal intensity might be due to the silica shell, which affected the average size and density of Ag NPs on the surface of GO or GO@SiO_2_. In particular, Ag NPs on thin silica-coated GO had a higher density than those of non-silica-coated GO. As a result, more 4-MBA can be adsorbed on the surface of GO@SiO_2_@Ag NPs, leading the higher intensity of GO@SiO_2_@Ag NPs compared to that of GO@Ag NPs. Furthermore, the thin silica shell allowed 4-MBA close to the GO@SiO_2_@Ag NPs to enhance the SERS signal both from Ag NPs to Ag NPs and from Ag NPs to the GO platform [72]. In order to confirm the importance of the thin layer of silica shell, we also prepared a thick layer of silica coated GO by adding 30 μL TEOS into the solution after treatment with sodium silicate. Figure S4 displayed the UV spectrum and SERS signal of thick layer of silica coated graphene oxide (thick GO@SiO_2_@Ag NPs). While the UV spectrum of thick GO@SiO_2_@Ag NPs was slightly reduced, the SERS signal was dramatically decreased. The SERS signals at 1072 cm^−1^ and 1582 cm^−1^ were 182 cps and 286 cps, respectively. This result was in good agreement with the previous reports that thick silica coating decreased the SERS enhancement compared to thin silica coating [57,58] due to the electromagnetic fields generated by Ag NPs decaying exponentially upon their distance from the core surface [73].

We next evaluated the SERS activity of GO@SiO_2_@Ag NPs. After introducing 4-MBA onto GO@SiO_2_@Ag NPs, the resulting material (0.1 mg/mL in ethanol) solution was drop-cast on a patterned glass slide to measure the SERS signal from single GO@SiO_2_@Ag NPs. After mapping the SERS signals with a 1 μm step size for 2 s using a 532 nm laser line, the Raman images were overlaid with the corresponding optical images. As shown in Figure S5a, the area of intense signals in the SERS intensity map corresponded to the position of the single GO@SiO_2_@Ag NPs in the optical micrographs, indicating that although SERS signals from various GO@SiO_2_@Ag NP sheets were dramatically different, the SERS signal was strong enough for the detection of each GO sheet (Figure S5b). Unfortunately, the SERS signals of single-sheet GO@SiO_2_@Ag NPs were highly variable, thus, we cannot use this technique for the quantitative detection of 4-MBA.

**Figure 4:**
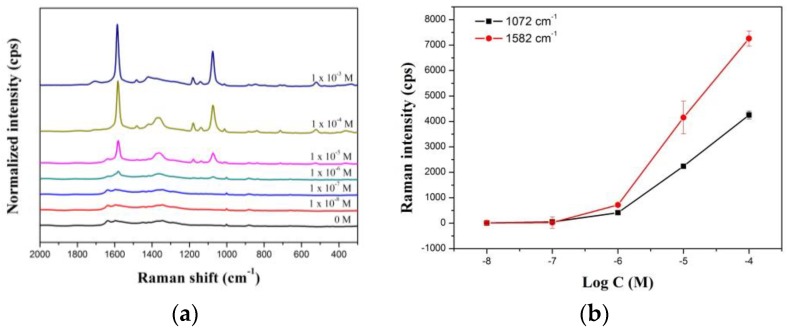
(**a**) SERS spectra and (**b**) calibration curves of GO@SiO_2_@Ag NPs to different 4-mercaptobenzoic acid in EtOH solution. Laser power is 10 mW, wavelength is 532 nm, integration time is 5 s, and the laser spot is 2 µm.

We also used GO@SiO_2_@Ag NPs as an active SERS substrate for the detection of 4-MBA in a solution as a model study. The GO@SiO_2_@Ag NPs were mixed with various concentrations of 4-MBA in ethanol. Figure 4a shows strong SERS signals of 4-MBA at concentrations of 1 × 10^−3^ to 1 × 10^−8^ M, confirming that the GO@SiO_2_@Ag NPs can be used to detect 4-MBA. Specifically, the intensities of the Raman bands at 1072, 1142, 1175, 1370, 1432, 1582, and 1710 cm^−1^ increased as 4-MBA concentration increased. The dominant increase was at 1072 and 1582 cm^−1^. Figure 4b shows the correlation between peak intensity and 4-MBA concentration. To verify the sensitivity of the GO@SiO_2_@Ag NP based detection, we calculated the theoretical LOD to detect 4-MBA at the 1582 cm^−1^ peak, based on the standard deviation of the response and the slope of the calibration curve at levels approximating the LOD according to the formula LOD = 3.3(standard deviation/slop). The theoretical LOD of the SiO_2_@Au@Ag NPs was 0.74 μM. The LOD of GO@SiO_2_@Ag NPs was higher than the LOD of GO@Ag NPs reported by Liu and Qian [62,74,75] but lower than GO-porphyrin materials [76]. The reproducibility of our materials was calculated from the measurement of 10 samples, as shown in Figure S6 (Supplementary Materials). The reproducibility was 9.1% after synthesis.

To investigate the stability of our materials, GO@SiO_2_@Ag NPs was redispersed in ethanol solution and stored at room temperature for seven days. The UV intensity of GO@SiO_2_@Ag NPs during these seven days was evaluated and plotted in Figure S7a (Supplementary Materials). The result showed that the UV intensity varied in the range of 100%–101%, indicating that GO@SiO_2_@Ag NPs were not aggregated for one week. To confirm the effect of Ag oxidation during storage on the SERS signal of GO@SiO_2_@Ag NPs, 10 μM 4-MBA (1 mL) was incubated with GO@SiO_2_@Ag NPs and measured Raman spectroscopy. The result is shown in Figure S7b. The SERS signal maintained its 60%–65% original value after six days due to the oxidation of Ag NPs.

## 3. Materials and Methods

### 3.1. Materials

Tetraethylorthosilicate (TEOS), 3-mercaptopropyl trimethoxysilane (3-MPTS), ethylene glycol (EG), polyvinylpyrrolidone (PVP), silver nitrate (AgNO_3_, 99.99%), octylamine, sodium silicate solution, and 4-mercaptobenzoic acid (4-MBA) were purchased from Sigma-Aldrich (St. Louis, MO, USA) and used without further purification. Nano graphene oxide (GO) was purchased from Graphene Supermarket (Calverton, New York, NY, USA). Ethyl alcohol (EtOH), and aqueous ammonium hydroxide (NH_4_OH, 27%) were purchased from Daejung (Siheung, Korea).

### 3.2. Method

#### 3.2.1. Thiolated Silica Coating on Graphene Oxide (GO@SiO_2_)

GO (6 mg) was dispersed into 15 mL water and 15 μL sodium silicate solution (0.036 wt%) and stirred vigorously for 12 h at 25 °C. Silica-coated GO was obtained by centrifugation at 12,000× *g* for 30 min and several washes with EtOH.

Silica-coated GO (3 mg) was dispersed into 10 mL EtOH. 3-MPTS (10 µL) and NH_4_OH (40 µL) was added to the dispersed solution and stirred vigorously for 6 h at 25 °C. Thiolated silica-coated GO (GO@SiO_2_) was obtained by centrifuging at 12,000× *g* for 30 min and several washes with EtOH.

#### 3.2.2. Preparation of Silver Nanoparticle-Embedded Silica-Coated Graphene Oxide (GO@SiO_2_@Ag NPs)

Ag NPs were introduced onto the surface of GO@SiO_2_ by the previously-reported method with some modifications [56,61]. Briefly, 2 mg GO@SiO_2_ was dissolved in 10 mL EG containing 2 mg PVP, followed by the addition of 10 mL AgNO_3_ solution (1 mg/mL in EG), and thoroughly mixed. Octylamine (41.3 µL) was added to the solution and the resulting suspension was stirred for 6 h at 25 °C. The nanoparticles were centrifuged at 5000× *g* for 15 min and washed several times with EtOH.

For comparison, GO without a silica coating was used as a control. Ag NPs were introduced onto the surface of GO by the aforementioned procedure. Silver nanoparticle-embedded graphene oxide was termed as GO@Ag NPs.

#### 3.2.3. Incorporation of 4-mercaptobenzoic Acid (4-MBA) into GO@SiO_2_@Ag NPs

4-MBA in EtOH (1 mL; 1 mM) was added to 1 mg of GO@SiO_2_@Ag NPs or GO@Ag NPs. The suspension was incubated for 1 h at 25 °C. The colloids were centrifuged and washed five times with EtOH. The materials were redispersed in EtOH to obtain 4-MBA-incorporated GO@SiO_2_@Ag or 4-MBA-incorporated GO@Ag solution (1 mg/mL).

### 3.3. Instrument

To evaluate the sensitivity of the synthesized SERS substrates, the SERS signals were measured in a capillary tube using a DXR™ Raman Microscope system (Thermo Fisher Scientific, Waltham, MA, USA. The SERS signals were collected in a back-scattering geometry using a 10× objective lens. A 532 nm diode-pumped solid-state laser was used as the photo-excitation source, with 10 mW laser power at the sample. Selected sites were measured randomly, and all SERS spectra were integrated for 5 s. The spot size of the laser beam was ~2 μm.

## 4. Conclusions

In conclusion, we developed Ag NP-embedded thin silica-coated graphene oxide (GO@SiO_2_@Ag NPs) nanomaterials as a SERS substrate. The 6-nm layer of silica was coated on the GO surface by sodium silicate and 3-MPTS. Afterwards, Ag NPs were introduced to the surface of silica-coated GO by silver nitrate reduction. The average diameter of Ag NPs was 58 ± 18 nm. The presence of the thin silica layer on the surface of GO@SiO_2_@Ag NPs enhanced the SERS signal of 4-MBA was ~1.8-fold compared to that in the absence of a silica layer. The GO@SiO_2_@Ag NPs exhibited the LOD of 4-MBA at 0.74 μM. The GO@SiO_2_@Ag NPs can be utilized as a highly sensitive SERS substrate for detection of trace amounts of chemicals in various fields.

## Supplementary Materials

The following are available online at www.mdpi.com/2079-4991/6/10/176/s1.

## Abbreviations

The following abbreviations are used in this manuscript:

NPs	Nanoparticles
SERS	Surface-enhanced Raman scattering
TEM	Transmission electron microscope
LOD	Limit of detection
AgNPs	Silver nanoparticles
3-MPTS	3-mercaptopropyl trimethoxysilane
TEOS	Tetraethylorthosilicate
4-MBA	4-mercaptobenzoic acid
GO	Graphene oxide
GO@SiO_2_	Thiolated silica-coated graphene oxide
GO@Ag NPs	Silver-embedded graphene oxide
GO@SiO_2_@Ag NPs	Silver-embedded thin silica-coated graphene oxide
