# Supplementary material for: Silver Nanoparticle-Embedded Thin Silica-Coated Graphene Oxide as an SERS Substrate
Source: Nanomaterials (Basel). 2016 Sep 22;6(10):176. doi: 10.3390/nano6100176 (PMC5245191; doi:10.3390/nano6100176)
Supplement: Supplementary file 1 [file nanomaterials-06-00176-s001.pdf]

Supplementary Materials: Silver Nanoparticle-Embedded Thin Silica-Coated Graphene Oxide as a SERS Substrate

Xuan-Hung Pham, Eunil Hahm, Hyung-Mo Kim, Seongbo Shim, Tae Han Kim, Dae Hong Jeong, Yoon-Sik Lee and Bong-Hyun Jun

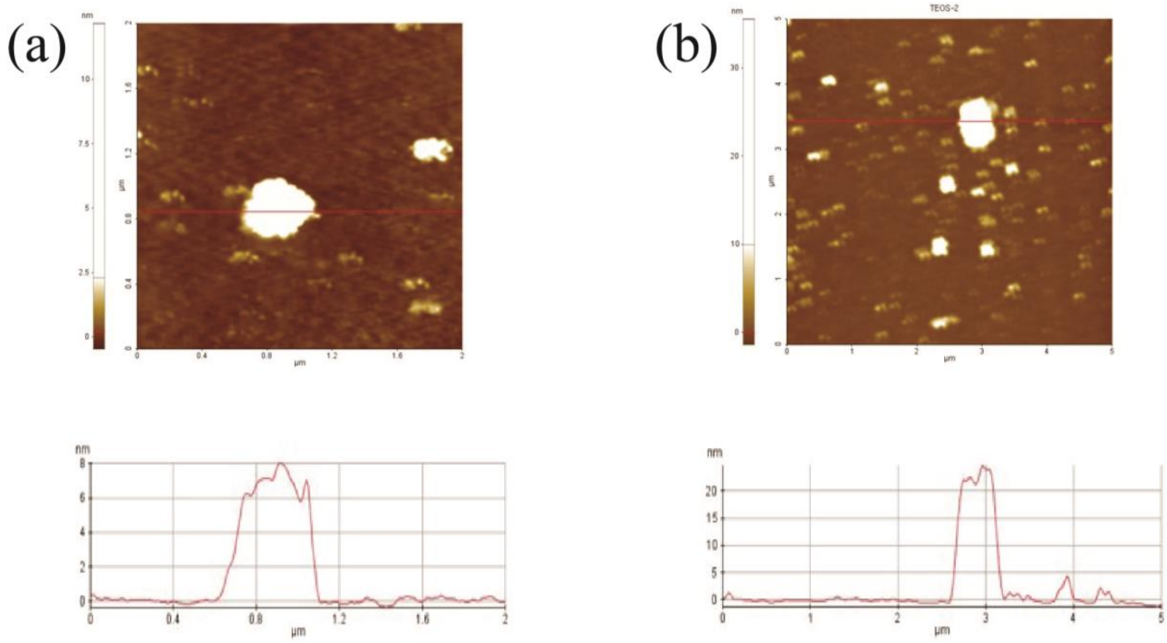

Figure S1. Atomic force microscopy images and histogram of (**a**) graphene oxide and (**b**) silica-coated GO. GO concentration is 1 mg/mL.

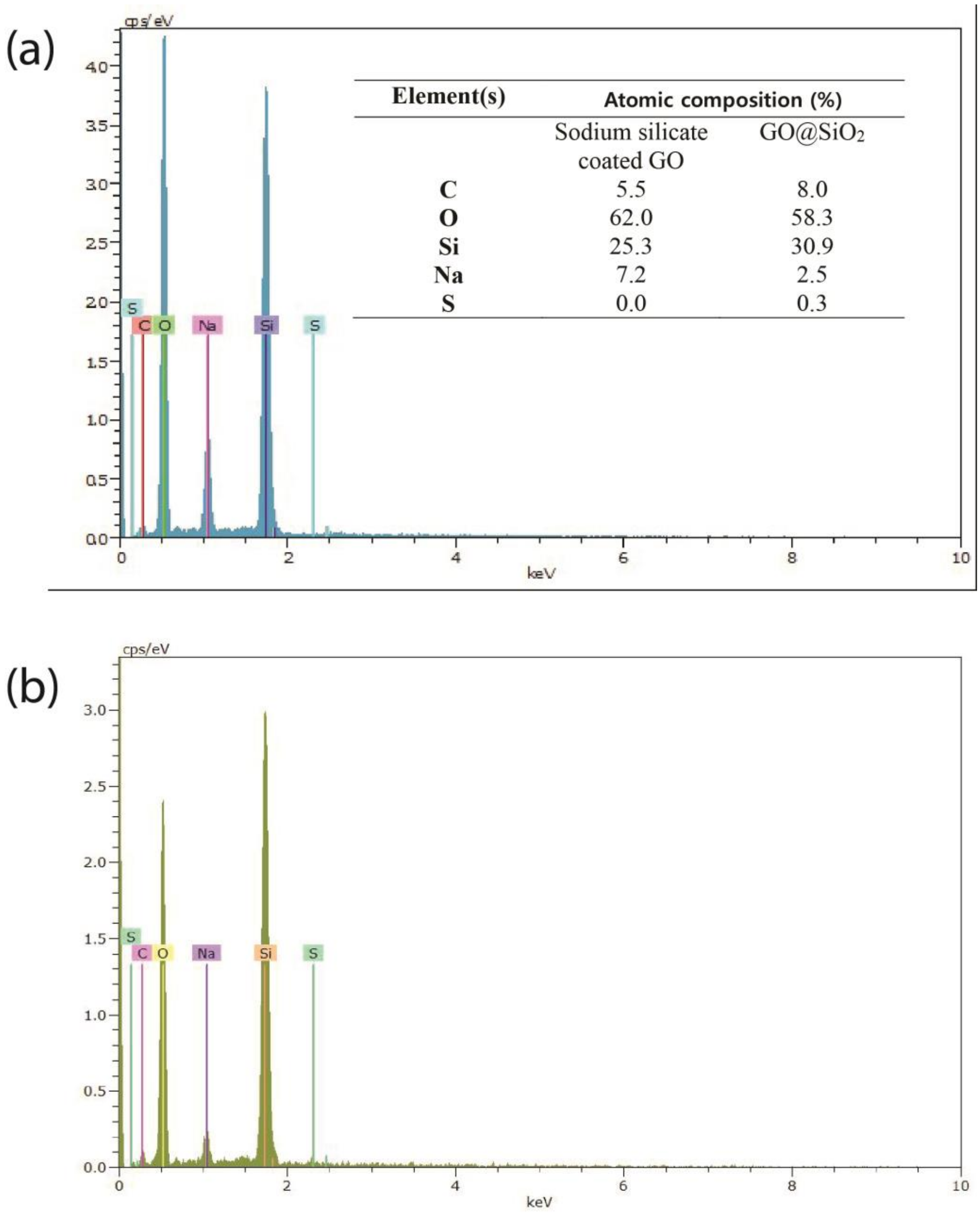

Element(s)	Atomic composition (%)	
	Sodium silicate coated GO	$GO@SiO_2$
**C**	5.5	8.0
**O**	62.0	58.3
**Si**	25.3	30.9
**Na**	7.2	2.5
**S**	0.0	0.3

Figure S2. Energy dispersive X-ray spectroscopy data of (**a**) sodium silicate coated graphene oxide and (**b**) thiol fuctionalized silica coated graphene oxide ($GO@SiO_2$).

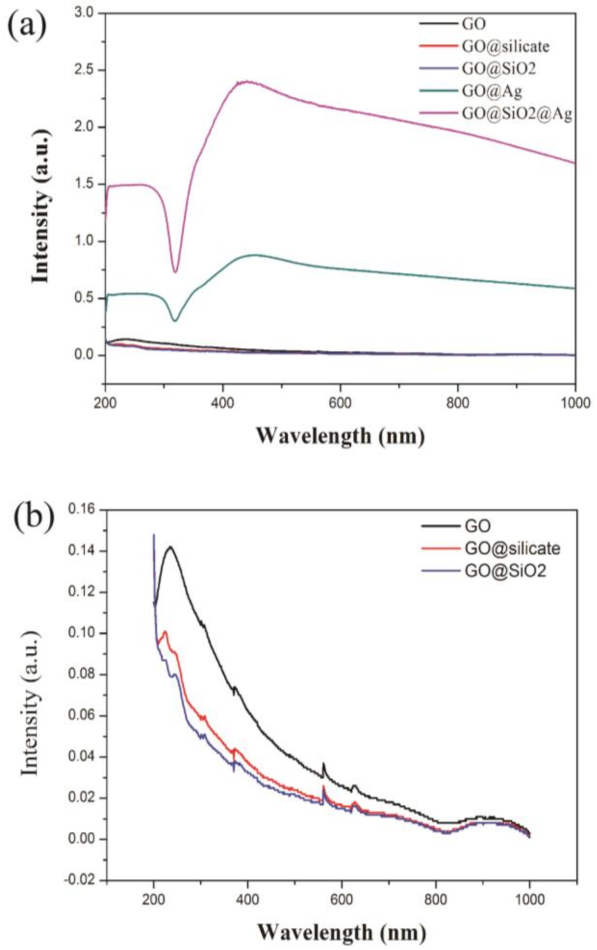

Figure S3. UV-vis spectroscopy (**a**) and enlargement (**b**) of graphene oxide (GO), silica-coated graphene oxide (GO@silicate), thiolated silica-coated graphene oxide (GO@SiO_2), silver nanoparticle-embedded silica-coated graphene oxide (GO@SiO_2@Ag) and silver nanoparticle-embedded graphene oxide (GO@Ag).

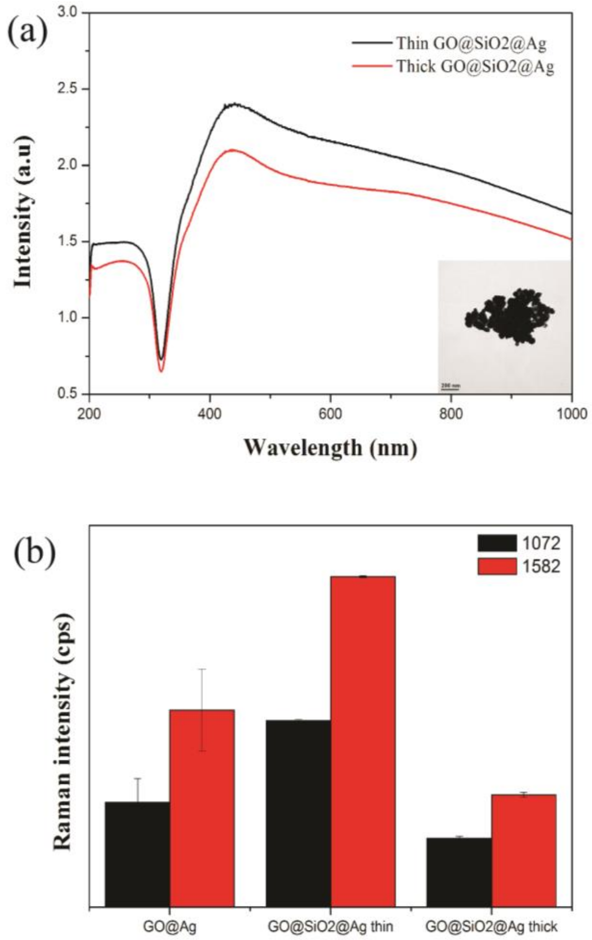

Figure S4. (**a**) UV spectra and (**b**) SERS intensity of thin and thick GO@SiO_2@Ag NPs in EtOH solution with 1 mM 4-mercaptobenzoic acid. Inset is TEM image of thick GO@SiO_2@Ag NPs. GO concentration is 1 mg/mL, laser power is 10 mW, wavelength is 532 nm, integration time is 5 s, and laser spot is 2 μm.

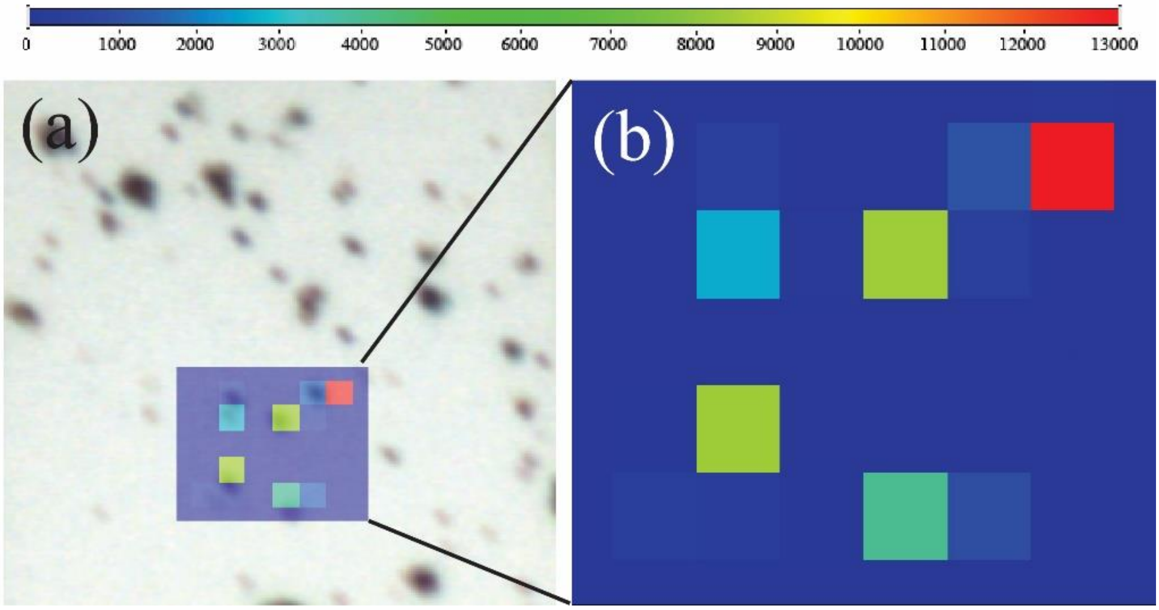

Figure S5. (**a**) Merged image of 2D Raman mapping and optical images of GO@SiO_2@Ag NPs with 1 mM 4-mercaptobenzoic acid. (**b**) 2D Raman mapping of GO@SiO_2@Ag NPs. GO concentration is 1 mg/mL. Laser power is 10 mW, wavelength is 532 nm, integration time is 5 s, and laser spot is 2 μm.

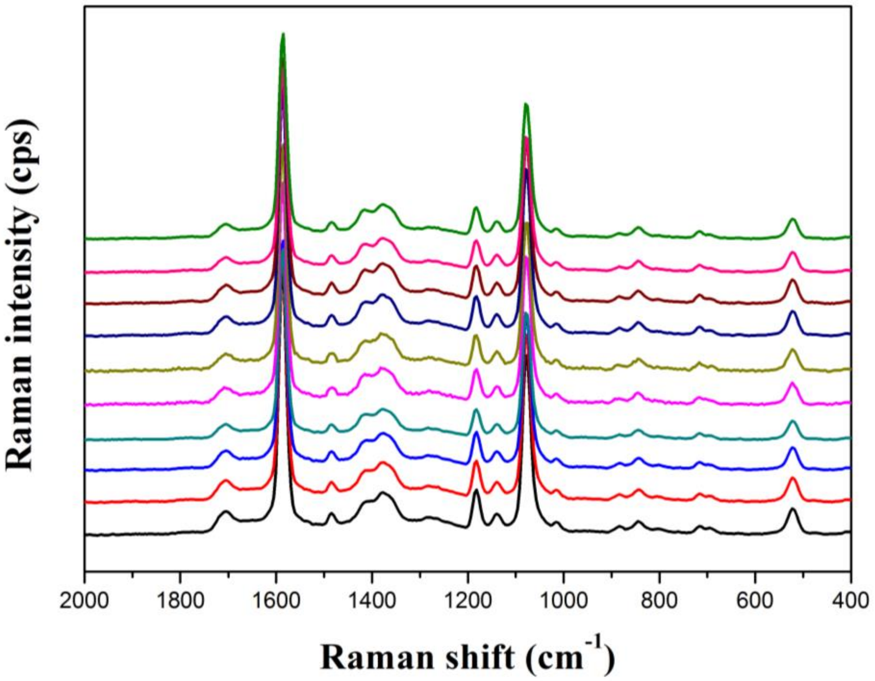

Figure S6. SERS spectra of 10 μM 4-MBA in ethanol solution contain GO@SiO_2@Ag NPs (1 mg/mL). The spectra range from 400–2000 nm.

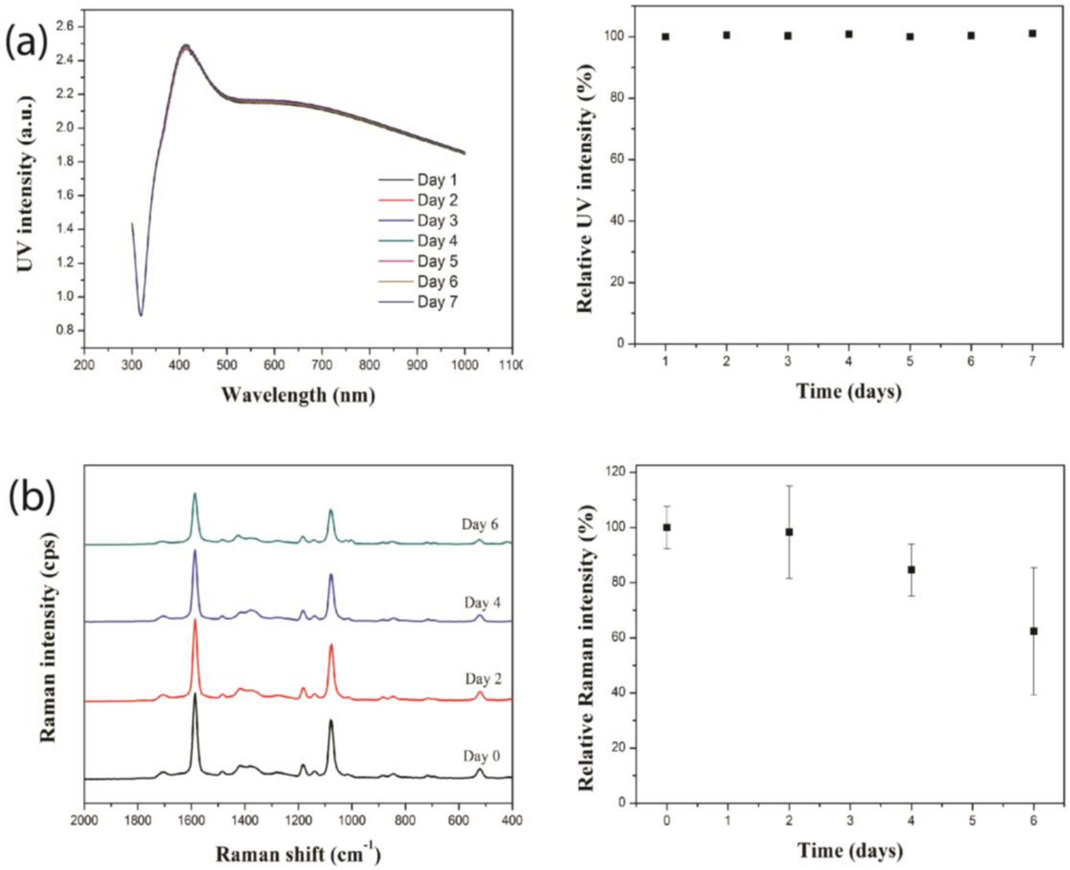

Figure S7. (**a**) UV spectra and (**b**) SERS spectra of GO@SiO_2@Ag NPs which stored in ethanol solution at room temperature in darkness. The spectra range from 400–2000 nm. Herein 10 μM 4-MBA (1 mL) was incubated with GO@SiO_2@Ag NPs and measured Raman spectroscopy.
